# Elastin is Localised to the Interfascicular Matrix of Energy Storing Tendons and Becomes Increasingly Disorganised With Ageing

**DOI:** 10.1038/s41598-017-09995-4

**Published:** 2017-08-30

**Authors:** Marta S. C. Godinho, Chavaunne T. Thorpe, Steve E. Greenwald, Hazel R. C. Screen

**Affiliations:** 1 Institute of Bioengineering, School of Engineering and Materials Science, Queen Mary University of London, London, E1 4NS United Kingdom; 2 Comparative Biomedical Sciences, The Royal Veterinary College, Royal College Street, London, NW1 0TU United Kingdom; 3 Blizard Institute, Barts and London School of Medicine and Dentistry, Turner Street, London, E1 11BB United Kingdom

## Abstract

Tendon is composed of fascicles bound together by the interfascicular matrix (IFM). Energy storing tendons are more elastic and extensible than positional tendons; behaviour provided by specialisation of the IFM to enable repeated interfascicular sliding and recoil. With ageing, the IFM becomes stiffer and less fatigue resistant, potentially explaining why older tendons become more injury-prone. Recent data indicates enrichment of elastin within the IFM, but this has yet to be quantified. We hypothesised that elastin is more prevalent in energy storing than positional tendons, and is mainly localised to the IFM. Further, we hypothesised that elastin becomes disorganised and fragmented, and decreases in amount with ageing, especially in energy storing tendons. Biochemical analyses and immunohistochemical techniques were used to determine elastin content and organisation, in young and old equine energy storing and positional tendons. Supporting the hypothesis, elastin localises to the IFM of energy storing tendons, reducing in quantity and becoming more disorganised with ageing. These changes may contribute to the increased injury risk in aged energy storing tendons. Full understanding of the processes leading to loss of elastin and its disorganisation with ageing may aid in the development of treatments to prevent age related tendinopathy.

## Introduction

Tendons provide an attachment from muscle to bone, playing an important role in locomotion. Their primary function is to transfer the force generated by muscle contraction to the skeleton, facilitating movement around joints and positioning the limbs^1, 2^. For efficient function, tendons must be strong and stiff under uniaxial tension, but also incorporate some viscoelasticity to optimize their stiffness to meet different loading conditions^3, 4^. These properties are conferred by the composition of tendon and the interaction of structural molecules throughout the hierarchical organisation of the extracellular matrix. In addition to transferring force, some tendons have an additional role of energy storage^1^. This function is critical for managing energy expenditure in humans and animals, and tendons performing this function are known as energy storing tendons^5^. The high strains these tendons must withstand during normal use make them more prone to highly debilitating and painful injuries termed tendinopathies, when compared to tendons that function purely to position the limb (positional tendons)^6^. Humans and horses have a similar mechanism for initiation and progression of tendon injury^7, 8^, with injury risk known to increase with age in both species. For this reason, the horse is commonly used as a model to study age related changes in tendon tissue^9^.

Tendons possess a highly organised structure, in which the subunits (collagen-rich fascicles) are oriented in the direction of force application, and surrounded by interfascicular matrix (IFM, sometimes referred to as the endotenon), which facilitates sliding between fascicles^10^. Energy storing tendons, such as the equine superficial digital flexor tendon (SDFT) and human Achilles tendon are more elastic and extensible than positional tendons, such as the equine common digital extensor tendon (CDET) and human anterior tibialis tendon. Pulling adjacent fascicles apart to test the IFM in shear^10^, Thorpe *et al*. have shown that energy storing tendons have a specialised IFM that exhibits low stiffness and fatigue resistance^10^, suggesting that it may enhance the elastic behaviour of the whole tendon^10^, by allowing it to stretch and recoil efficiently.

With ageing, the IFM becomes stiffer and less fatigue resistant, especially in energy storing tendons^11^, which may explain why these tendons become more prone to injury as they age. Taken together, data suggest that the IFM plays a crucial role in tendon mechanical properties, particularly in energy storing tendons. However, interfascicular matrix composition remains poorly defined. Previous studies have shown that the IFM has a distinct composition and greater cell density compared to fascicles^12^. Fascicles consist of highly aligned collagen fibres interspersed with sparsely distributed elongated cells, whilst the IFM is more disorganised in appearance, with a greater number of more rounded cells, more collagen type III and proteoglycans (particularly lubricin), but a lower collagen type I content^12–14^. Additionally, recent work suggests a greater rate of matrix turnover within the IFM, which may act to maintain healthy tendon structure^12^.

Several recent studies have reported that the IFM is also rich in elastin^15–17^, where it may influence IFM and consequently whole tendon mechanics. Elastin is the core protein of elastic fibres and abundant in repetitively loaded tissues such as arteries^18^, lungs^19^ and skin^20^ where it provides extensibility and resilience to the extracellular matrix^21^. Tropoelastin is the soluble, monomeric form of elastin secreted by cells; its structure consists of alternating hydrophobic and crosslinked domains^22, 23^. The process responsible for the formation of functional elastin within the elastic fibre is known as elastogenesis. Tropoelastin molecules are synthesised and assembled into microfibrils in a process mediated by the glycoprotein fibrillin^24^, which can then be crosslinked by lysyl oxidase, resulting in the formation of the stable and non-reducible tetrafunctional desmosine and isodesmosine crosslinks^25–27^. The highly crosslinked nature of mature elastin means it is insoluble and highly resistant to proteolytic degradation, making it one of the most stable proteins in the body. However, under repetitive loading, it undergoes fragmentation which accumulates over time, thus affecting its function^28^.

Overall elastin content in tendon is low and it is sparsely distributed within fascicles^13^. Data indicating its localisation to the IFM^15^, suggest it may be particularly important for IFM function, although a quantitative assessment of elastin localisation has yet to be carried out. There are no studies reporting elastin content, organisation or changes with ageing in tendons with different functions. Therefore, in this study we aimed to quantify the total amount of elastin in different tendon types and age groups, as well as to determine elastin organisation and fragmentation in both fascicular matrix (FM) and IFM and how this changes with ageing. We hypothesised that elastin is more prevalent in energy storing than positional tendons, and that it is mainly localised to the IFM. Further, we hypothesised that elastin becomes disorganised and fragmented, and decreases in amount with ageing, especially in energy storing tendons.

## Results

### Tendon elastin quantification

**Figure 1:**
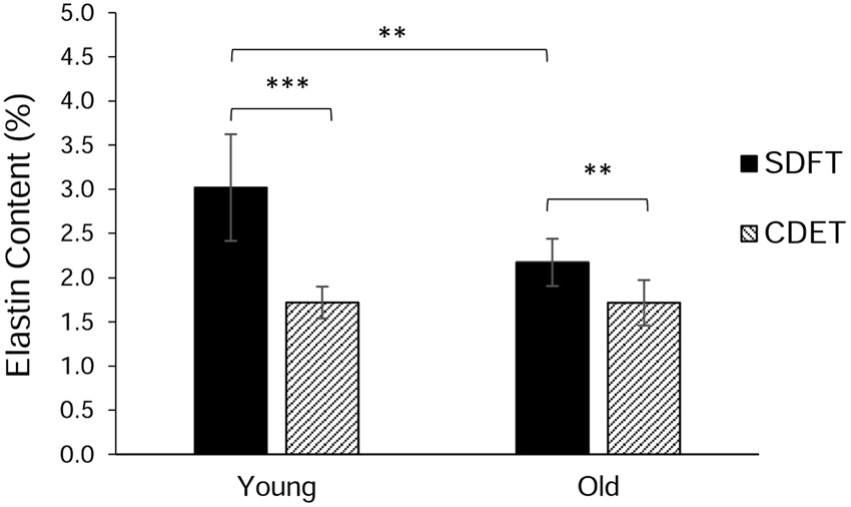
Elastin content expressed as a percentage of dry weight of tendon tissue from 14 horses aged 3 to 7 years (defined as young group, n = 7) and 15 to 19 years (old group, n = 7). Significant differences between tendon types and age groups are identified by: **p < 0.01 and ***p < 0.001. In this and subsequent figures data are displayed as mean ± standard deviation.

Tendon elastin content was determined using the FASTIN elastin assay (Biocolor, UK). Results showed that elastin content in the whole tendon tissue was significantly greater in the energy storing SDFT (3.02% ± 0.60) than in the positional CDET (1.72% ± 0.18, p < 0.001) (Fig. 1). With age, the amount of elastin decreased in the SDFT (2.17% ± 0.27, p < 0.01) whereas there were no age related changes in elastin content in the positional CDET (Fig. 1).

### Desmosine Quantification

**Figure 2:**
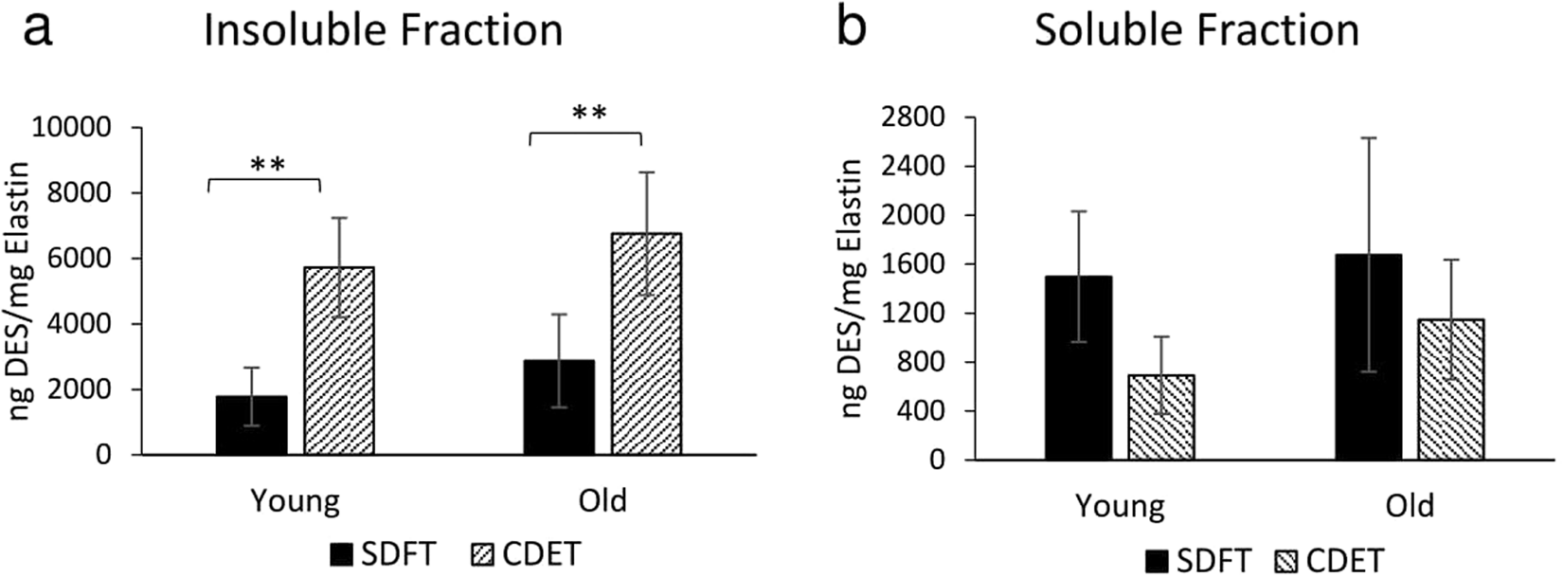
Desmosine content expressed as ng desmosine per mg of elastin in both insoluble (**a**) and soluble (**b**) fractions of SDFTs and CDETs from horses separated into 2 groups based on age (n = 6/age group). Note the differences in scale in the y axis between **a** and **b**. Significant differences between tendon types and age groups are identified by: **p < 0.01.

Desmosine, one of the mature elastin crosslinks, was used as a marker of elastin fragmentation^29–32^. Tendon samples were separated into soluble and insoluble fractions using guanidine hydrochloride extraction and the amount of desmosine in each fraction was determined. Whilst the desmosine in the insoluble fraction represents the functional elastin crosslinked within the tendon matrix, the desmosine in the soluble fraction represents the crosslinked mature elastin which has been partially degraded. In the insoluble fraction, the amount of desmosine was significantly greater in the CDET (5720.8 ng DES/mg elastin ± 1516.8) compared to the SDFT (1776.1 ng DES/mg elastin ± 888.6, p < 0.01) (Fig. 2a). However, no tendon type differences were seen in the soluble fraction (Fig. 2b). Similarly, in the old group, the amount of desmosine was significantly greater in the CDET (6758.5 ng DES/mg elastin ± 1875.4) compared to SDFT (2871.8 ng DES/mg elastin ± 1418.9, p < 0.01) (Fig. 2a) with no tendon type differences identified in the soluble fraction (Fig. 2b). No age-related differences in desmosine content were seen, either in the soluble or insoluble fractions.

### Tendon elastin images semi-quantification

**Figure 3:**
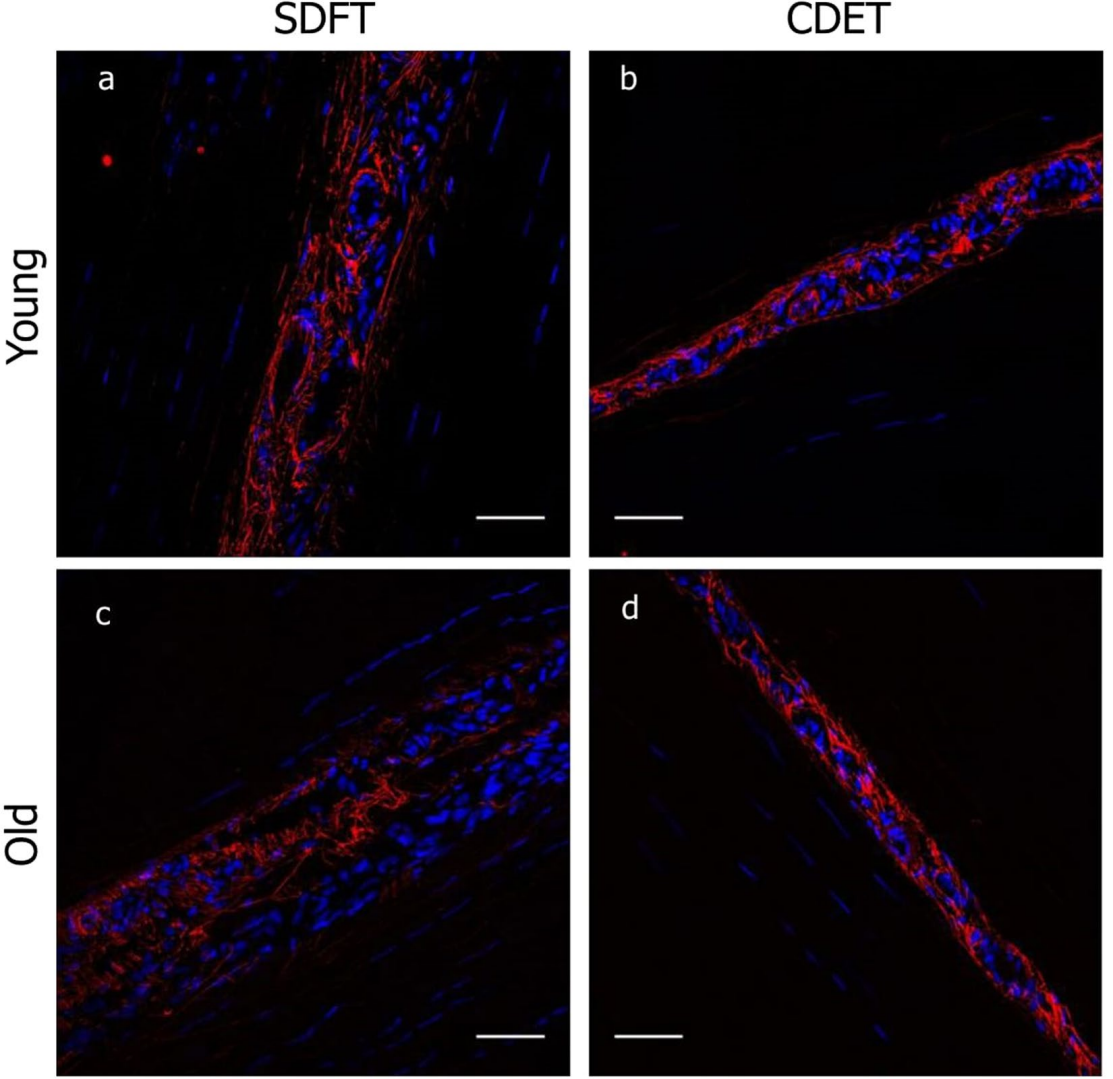
Representative confocal images showing elastin (red) organisation in young and old SDFT and CDET. Longitudinal sections from young SDFT (**a**), young CDET (**b**), old SDFT (**c**) and old CDET (**d**) were immunostained for elastin (red). Cell nuclei were also stained (blue). Scale bar: 50 μm.

**Figure 4:**
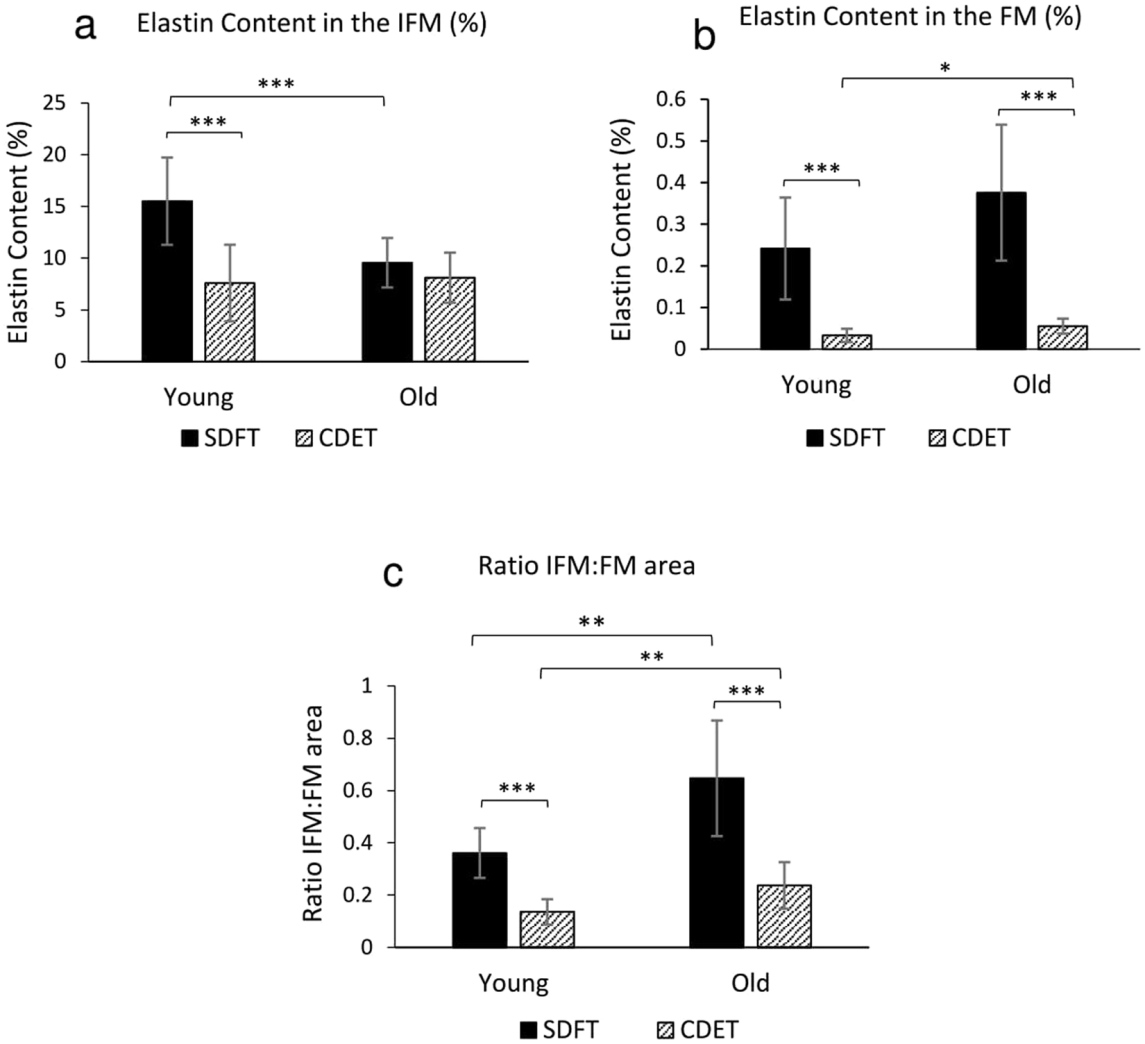
Percentage area of IFM (**a**) and FM (**b**) showing positive immunostaining for elastin in young and old SDFTs and CDETs. Note the differences in scale between the IFM (**a**) and FM (**b**) regions. The ratio of IFM:FM area was also calculated for all samples, and is compared across tendon types and age groups (**c**). Significant differences between tendon types and age groups are identified by: *p < 0.05, **p < 0.01 and ***p < 0.001.

The amount of elastin in young and old SDFTs and CDETs was also semi-quantitively analysed as a percentage coverage within both the IFM and FM of histological sections immunostained for elastin. Results showed that most of the elastin (>90%) was localised to the IFM in both tendon types (Fig. 3). Comparing tendon types, the SDFT IFM showed greater immunostaining for elastin (area fraction: 15.5% ± 4.2) than the CDET IFM (7.6% ± 3.7, p < 0.001) (Figs. 3a,b and 4a). Similarly, the SDFT FM showed a greater percentage of elastin staining (0.24% ± 0.12) than the CDET FM (0.03% ± 0.02, p < 0.001) (Figs 3a,b and 4b).

With ageing, there was a reduction in the amount of elastin in the IFM of the energy storing SDFT (9.6% ± 2.4, p < 0.001), however no age-related changes were seen in the CDET IFM (Figs 3b,d and 4a). In the FM, there was a slight increase with ageing (p = 0.038) in the percentage of elastin staining in the positional CDET and no age-related changes in the SDFT (Fig. 4b). There was also a significantly greater IFM:FM area ratio in the SDFT (0.36 ± 0.10) compared to the CDET (0.14 ± 0.05, p < 0.001), and this ratio increased with age in both tendon types (SDFT: 0.64 ± 0.22, p < 0.01 and CDET: 0.24 ± 0.09, p < 0.01) (Fig. 4c).

To determine if the elastin within tendon is localised to the vicinity of blood vessels, a subset of SDFT and CDET cryosections were stained for both elastin and the endothelial cell marker CD31. It was notable that CD31 staining was observed in all samples. However, staining consistently detected only very low levels of CD31, which were always localised exclusively to the IFM region (Supplementary Fig. 1), indicating that the majority of elastin within tendon is not associated with the presence of blood vessels.

### Tendon Elastin Organisation

#### Fast Fourier Transform

**Figure 5:**
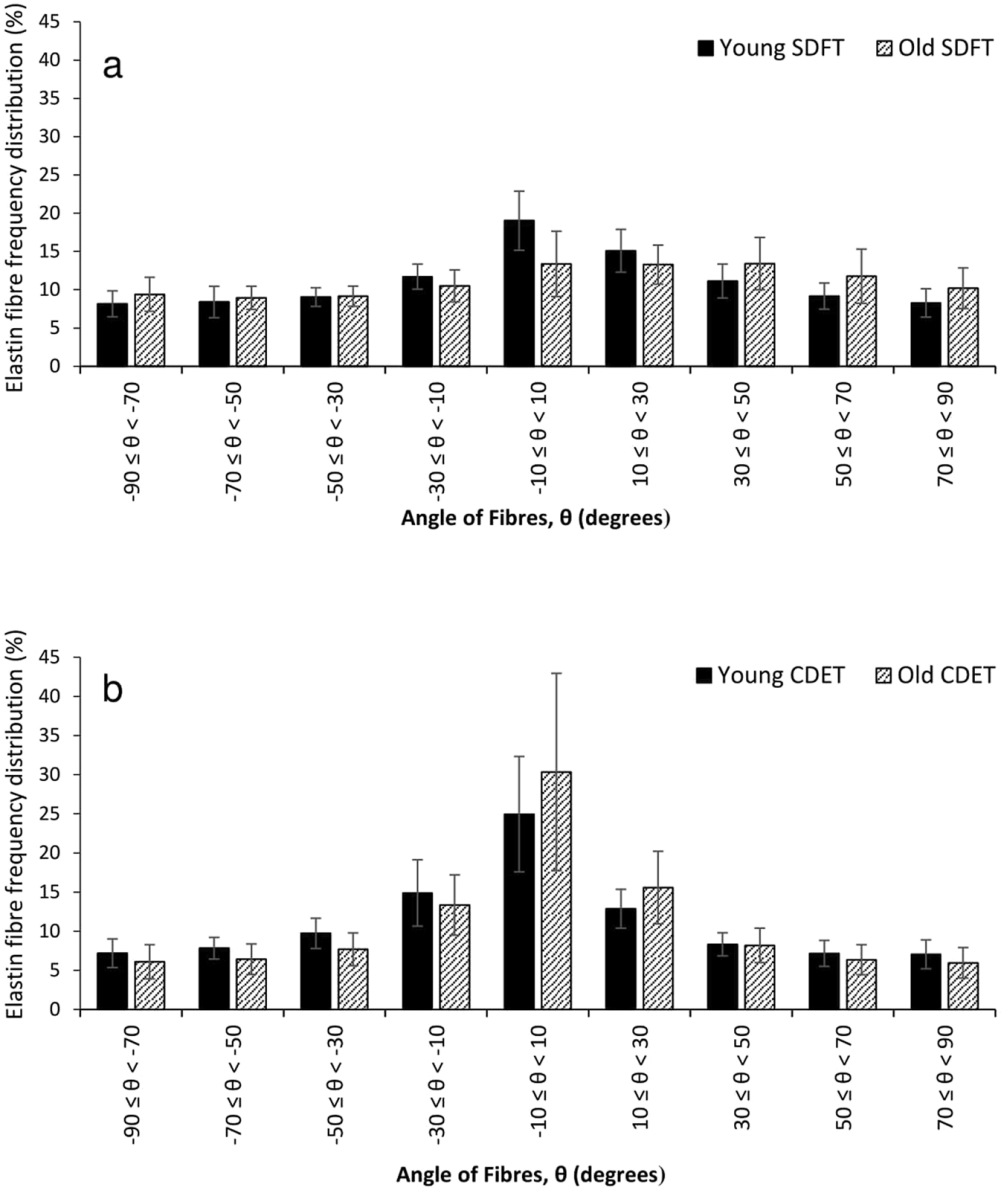
Frequency distribution of elastin fibre alignment, comparing young and old SDFT samples (**a**) (n = 3), and young and old CDET samples (**b**) (n = 3), with data combined from 48 images. Angles (θ) are given relative to the long axis of the tendon (denoted as 0 degrees).

A Fourier transform was performed on the immunohistological images to assess differences in the orientation and alignment of elastin fibres between tendon types and age groups. Analyses of the frequency distribution of elastin fibre orientation in each image showed no significant differences in the circular standard deviation between tendon types in young samples, and no age related changes in CDETs (Table 1 and Fig. 5a and b). On the other hand, old SDFTs have a higher circular standard deviation compared to young SDFTs (Fig. 5a and Table 1), indicating that elastin fibres in energy storing tendons become more disorganised with age. Also, old SDFTs showed a higher circular standard deviation compared to old CDETs (Table 1), suggesting that the age related loss of elastin organisation is specific to the SDFT.

**Table 1:** Circular standard deviation values, denoting the variability in elastin organisation in young and old SDFTs and CDETs.

Circular Standard Deviation	Young	Old
SDFT	42.81 ± 2.86	46.10 ± 3.38*
CDET	40.37 ± 4.02	37.32 ± 5.52ª

A larger number denotes more randomly distributed fibres. Significant differences between young and old SDFTs are denoted by: *p < 0.05, whilst significant differences between old SDFTs and old CDETs are denoted by: ªp < 0.001. Data are displayed as Mean ± Standard Deviation.

#### Image scoring

**Figure 6:**
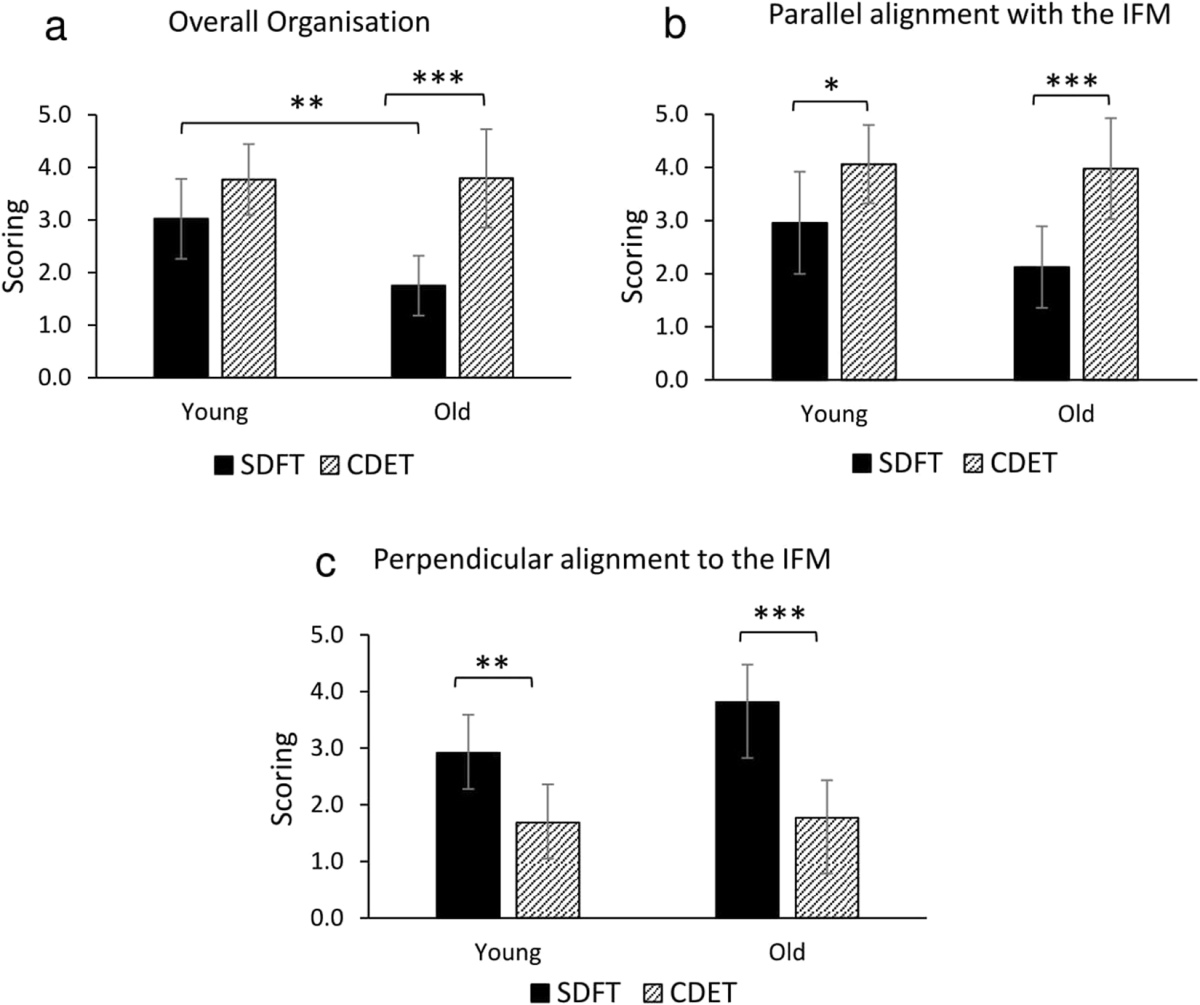
Image scoring for overall elastin organisation (**a**), alignment of elastin parallel with the IFM (**b**) and alignment of elastin perpendicular to the IFM (**c**). Overall elastin organisation within the IFM was scored on a scale from 1 to 5, where 1 denoted highly disorganised elastin and 5 denoted highly organised elastin. A scale of 1–5 was also used for elastin alignment parallel or perpendicular to the IFM, where 1 denotes poor alignment in the direction of interest and 5 denotes high alignment in that direction. Significant differences between tendon types and age groups are identified by: *p < 0.05, **p < 0.01 and ***p < 0.001.

Additional analysis of the immunohistological images was performed by blinded scorers, who assessed, with a 5-point scale, the overall organisation of elastin, and its orientation parallel and perpendicular to the IFM. Data indicated that in both young and old tendons, CDET elastin fibres showed significantly greater alignment parallel to the IFM (Fig. 6b), whilst SDFT elastin fibres showed a significantly greater alignment perpendicular to the IFM (Fig. 6c). Furthermore, there was a significant decrease in the overall elastin organisation with ageing in SDFTs, such that in old samples, SDFT images showed significantly reduced overall elastin organisation relative to old CDETs (Fig. 6a). The average pairwise Cohen’s Kappa for the overall elastin organisation, fibre alignment parallel to the IFM and perpendicular to the IFM were: 0.45, 0.48 and 0.39 respectively, where 0.21–0.40 is considered fair agreement, 0.41–0.60 moderate agreement, 0.61–0.80 substantial and 0.81–1 almost perfect agreement between assessors^33^.

## Discussion

This is the first study to quantify elastin content in two functionally distinct tendons, additionally reporting changes with ageing. The organisation and distribution of elastin fibres were also investigated, using several techniques to characterise their alignment, organisation and fragmentation in both young and old SDFTs and CDETs. The results broadly support our hypotheses, showing a greater amount of elastin in the energy storing SDFT, mainly localised to the IFM. With ageing, there was a decrease in elastin content and organisation in the energy storing SDFT, although no fragmentation was identified.

Most of the elastin (>90%) was localised to the IFM in both tendon types, with greater elastin staining observed in the SDFT IFM compared to the CDET IFM. These findings support previous studies that have shown the IFM is comparatively rich in elastin, both in tendon^15^ and ligament^17^. Further, our data confirm recently published work suggesting a greater abundance of elastic fibres in the IFM of the energy storing SDFT, relative to the CDET IFM^16^. Previous studies have shown that the IFM in the SDFT is more extensible and less viscoelastic compared to CDET IFM^10, 34^. Therefore, these data suggest that the presence of elastin may provide the IFM with its elastic recoil ability, necessary for the efficient energy storing and return that is required in energy storing tendons^34^.

It is important to highlight that this study is focused on equine tendon, and specifically on elastin as a component of the IFM. It is possible that energy storing tendons from other species utilise different mechanisms to facilitate extension, or that IFM components other than elastin confer the specialised mechanical properties of the IFM. However, the role of the SDFT, as an extreme energy storing tendon^1^, makes it a useful tendon to investigate, as we would expect specialisations to be more pronounced. Also, as we are investigating structural differences associated with fatigue resistance, an initial focus on elastin seems appropriate.

We should note that some of the elastin seen in the IFM may be associated with blood vessels. Indeed, the positive staining for endothelial markers observed in all samples of both tendon types, confirms the presence of blood vessels in both the SDFT and CDET, and localisation of vessels to the IFM, agrees with previous data identifying this region as the source of tendon vascularisation^35, 36^. The extent of CD31 staining is perhaps surprising, with every region of IFM in all samples staining positive, indicating that vascularisation of the IFM is extensive in all tendon types, not just the more highly loaded energy storing tendons. However, despite the prevalence of CD31 staining, a qualitative analysis of the IFM highlighted that the majority of elastin was not localised exclusively to the region of CD31 staining and vessels, suggesting a role for elastin as an integral component of the tendon matrix also. Applying a fast Fourier transform to the confocal images, yielded quantitative data about elastin alignment and orientation, and allowed comparison with a more traditional histological image scoring approach^37, 38^. In the young group, there were no significant differences in elastin organisation between tendon types, with both frequency distributions identifying a predominant fibre orientation parallel to the IFM. This seems at odds with the additional image analysis performed by the independent scorers, who reported that there were a greater number of elastin fibres aligned perpendicular to the IFM in the young SDFT than in the CDET. However, a closer comparison of the quantitative frequency distribution data does suggest that a greater proportion of fibres are fully aligned with the IFM in the CDET than the SDFT and fibres orientation is more evenly distributed in the SDFT. As fibres denoted “perpendicular” to the IFM by the scorers are unlikely to be at a precise angle of 90 degrees, it is likely the range of “perpendicular” fibres are seen across the bins at either end of the frequency distribution data, where greater values are seen for the SDFT compared to CDET. The presence of perpendicularly aligned elastin fibres in the SDFT IFM, suggests that, elastin orientation in the IFM may indeed play a crucial role in allowing fascicles to slide. We hypothesise that elastin fibres oriented in this perpendicular manner may serve as a “bridge” between adjacent fascicles, which would assist in returning fascicles to their initial location once external forces have been removed.

With ageing, circular standard deviation increased in the SDFT, resulting in a significant difference in elastin organisation between old SDFTs and CDETs. This suggests that elastin fibres in the IFM of CDETs tend to remain more closely aligned with the tendon long axis than those in the IFM of the SDFTs, which become more dispersed with age.

These data correlate well with image scoring, which indicated a significant decrease in the overall organisation of elastin in old SDFTs compared to young SDFTs. Taken together, the results indicate that elastin becomes more disorganised with ageing in the energy storing SDFT. Additionally, a significant increase in the IFM:FM area ratio in both tendon types was observed with ageing, suggesting that the IFM may be losing its structural integrity, possibly associated with changes in elastin organisation and decreased elastin content.

In this study, we have used immunofluorescent methods to assess semi-quantitatively the amount of elastin in different tendon compartments. Future studies should focus on establishing the absolute amounts of elastin within the IFM and FM in both tendon types. Previous analysis of the IFM and FM proteome did not identify elastin in either IFM or FM. However, is likely that this is because elastin is extremely insoluble and highly crosslinked, and therefore particularly difficult to identify using mass spectrometry^39^. These properties also mean elastin is highly resistant to proteolytic degradation. However, under repetitive loading, it undergoes damage which accumulates over time^28^. With ageing, elastin is also degraded through chemical mechanisms which affect its function^40^. Studies have shown an increase in elastin fragmentation with ageing, which links to changes in elasticity in cardiovascular tissues and increased human mortality^30, 41^.

Indeed, previous studies have demonstrated that the IFM stiffens with age and becomes less fatigue resistant in energy storing tendons^11, 42^. Elastin is a highly extensible, elastic and fatigue resistant protein, hence the overall reduction in IFM elastin seems likely to lead to reduced IFM extensibility. Further, the loss of elastin and associated reduction in IFM fatigue resistance in ageing tendons has previously been linked to reduced IFM recoil after loading^11, 42–44^, and current data indicates that this may stem for the loss of elastin organisation and reduction in elastin “bridges” between fascicles.

Taking these findings together, it was hypothesised that the reduction in elastin content and increased elastin disorganisation with ageing may be due to elastin fragmentation. Thus, in the current study, desmosine, an unique crosslink in mature elastin, was used as a marker of elastin fragmentation^29–32^. The number of desmosine crosslinks was measured in the insoluble fraction (functional elastin crosslinked within the tendon matrix), and also in the soluble fraction (crosslinked mature elastin which has been partially degraded and therefore can be solubilised^29^). Data showed a 2-fold greater amount of desmosine per unit elastin content in the insoluble fraction of CDETs compared to SDFTs, however, no tendon type differences were seen in the soluble fraction.

These results were unexpected and do not support our hypothesis. This might be due to the fact that the method used cannot detect fragmentation if the fragments remain insoluble. Alternatively, this might indicate that CDETs are more densely crosslinked compared to SDFTs. Previous studies have shown that the cross-linked regions of elastin are more rigid than regions free of crosslinks, meaning more crosslinking would reduce the overall compliance of the surrounding structure^45^. This may explain why CDET IFM is less extensible than SDFT IFM. Similarly, in the old group, the CDET has a greater number of desmosine crosslinks in the insoluble fraction compared to SDFT. In the soluble fraction, once again, no significant changes were identified. However, it is important to highlight the greater variation in the SDFT data, mainly in the soluble fraction, compared to CDET. This could be due to variations in exercise history between horses, which may lead to different levels of mechanical degradation within the tendon and different degrees of crosslink fragmentation. Unfortunately, it was not possible to collect an exercise history for the horses used in this study. It is also noteworthy that previous studies have demonstrated large variations in the mechanical properties of the IFM in the SDFT, particularly in the stiffness, failure properties^43^, and fatigue resistance^42, 44^. These individual variations may result in the large variations in SDFT failure properties reported previously^11, 46^ and also aid in explaining why some individuals are more at risk of tendon injury than others.

## Conclusions

This study demonstrates, for the first time, differences in elastin content in functionally different equine tendons and also identifies changes with ageing. The energy storing SDFT has a significantly greater elastin content than positional CDET, with most of the elastin localised to the IFM in both tendon types. These findings indicate that the presence of elastin may provide the IFM with its elastic recoil ability, necessary for efficient energy storage and return. With ageing, the reduction in elastin content of the SDFT IFM, may contribute to the previously observed stiffening and reduced resilience of the IFM in aged energy storing SDFTs, leading to age-related tendon injury. Differences in elastin crosslinking between tendon types may also contribute to the greater elasticity of both the SDFT IFM and the tendon as a whole in relation to the CDET.

## Methods

### Sample dissection and preparation

Forelimbs distal to the carpus were collected from horses aged 3 to 7 years (defined as young group, n = 7) and 15 to 19 years (old group, n = 7) euthanized at a commercial abattoir. Superficial digital flexor tendons (SDFTs) and common digital extensor tendons (CDETs) were harvested from each limb within 24 hours of death. Tendons were examined macroscopically to ensure there were no signs of injury. Six small samples, approximately 5 × 5 × 5 mm in dimension, were isolated from the mid-metacarpal region of every tendon: 2 for immunofluorescence and 4 for biochemical analyses. Samples intended for immunostaining were immediately embedded in optimal cutting temperature compound and snap frozen in hexane cooled on dry ice. Two longitudinal cryosections, 10 μm thick, were then cut from each sample and placed on poly-lysine slides, which were stored at −80 °C until required. The samples for biochemical analyses were snap frozen in liquid nitrogen and powdered (2 cycles of 3 minutes at 2000 rpm) using a Mikro Dismembranator (Sartorius, GE). Powdered samples were weighed, freeze dried over night and re-weighed to determine water content, and stored at −20 °C until required.

### Biochemical Analysis

#### Quantification of tendon elastin

The elastin content of native tendon tissue from young and old SDFTs and CDETs (n = 7) was quantified using the FASTIN^TM^ Elastin Assay (Biocolor, UK)^47, 48^. Before the assay could be performed, the native hydrophobic elastin in the powdered, lyophilised samples (~3 mg dry weight) required conversion into the water soluble derivate α-elastin. The conversion was achieved by heating the samples to 100 °C for 2 one hour periods with 750 µl of 0.25 M oxalic acid to guarantee complete solubilisation of the tissue elastin. Preliminary tests were performed to determine that two extractions were sufficient to solubilise all elastin. The solubilised elastin was directly assayed following the manufacturer’s instructions (Biocolour, UK), with α-elastin standards prepared using the 1 mg/ml α-elastin standard solution provided. Absorbance of samples and standards was determined in duplicate at 513 nm spectrophotometrically (SPECTROstar *Nano* microplate reader, BMG Labtech, Germany). Elastin concentration was calculated by comparison to the standard curve, and expressed as a percentage of tendon tissue dry weight.

### Desmosine Quantification

#### Protein extraction

Approximately 20 mg of lyophilised tissue was used from each sample (previously dissected from young and old SDFTs and CDETs (n = 6)), for desmosine quantification. Soluble proteins were extracted by incubating overnight with agitation at 4 °C in guanidine hydrochloride (GuHCl; 600 μl of 4 M guanidine hydrochloride, 50 mM sodium acetate, pH 6.8 plus protease inhibitor cocktail)^49^. The remaining pellet was centrifuged (15000 g for 15 minutes) and the supernatant collected, after which the pellet was re-suspended in GuHCl and the extraction process repeated. A solution of 50 mM sodium acetate in 95% ethanol was added to the supernatant, at a ratio of 900 μl per 100 μl of supernatant. The soluble fraction was precipitated overnight at −20 °C, and the supernatant was removed following centrifugation (16000 g for 25 min). The pellet was washed three times (to remove all guanidine) with 95% ethanol in water, air dried and re-dissolved in 600 μl of 50 mM sodium acetate. The insoluble fraction was washed six times with water and freeze dried for 24 h. Samples were weighed to determine how much soluble protein was extracted. Samples were then assayed for desmosine content as described below.

#### Desmosine quantification

A solid–phase competition Enzyme-linked Immunosorbent assay (ELISA) (B.I.T.S.® Desmosine ELISA Kit (Mologic Ltd, UK) was adopted to quantify levels of the crosslink desmosine as a marker of elastin degradation^29, 50, 51^ from 6 young and 6 old SDFTs and CDETs. First, total elastin content was measured in a small amount of both the soluble and insoluble fractions of each sample, using a FASTIN elastin assay (described above) (Biocolor, UK). Then, the remaining soluble and insoluble fraction of each sample was hydrolysed with 6 M HCl (5 mg/ml) for 48 h at 110 °C. Samples were neutralised by adding an equivalent amount of 6 M NaOH to every sample. Soluble and insoluble fractions were diluted prior to assay: 1:3 and 1:10, respectively to bring them within the range of the standard curve. The ELISA protocol was performed according to the manufacturer’s instructions (Mologic LTD, UK). Both standards and test samples were assayed in duplicate. Desmosine content was determined by comparison to a standard curve and expressed as ng desmosine per mg of elastin content.

### Assessment of tendon elastin organisation

#### Immunostaining

Longitudinal cryosections (n = 3) were allowed to thaw, before fixing with ice-cold methanol (−20 °C) for 20 minutes. Slides were rinsed 3 times in phosphate-buffered saline (PBS), treated with hyaluronidase (Sigma, H3884) (4800 units/ml in PBS containing protease inhibitor cocktail (complete mini, Roche)) to increase tissue permeability^52^, and incubated over night at room temperature. Sections were again washed 3 times in PBS and incubated in blocking buffer (10% goat serum in PBS) for 1 hour at room temperature. They were then drained and the elastin primary antibody (ab9519, Abcam Cambridge, UK) diluted in 5% goat serum in PBS (1:100 dilution) applied to each section before incubation overnight at 4 °C. After overnight incubation, sections were washed in PBS and incubated with Alexa Fluor 555 Goat anti-Mouse IgG Secondary Antibody diluted in 5% goat serum (1:500 dilution) for 1 h at room temperature (protected from light). Finally, sections were washed and mounted with ProLong Gold Antifade reagent with 4′,6-diamidino-2- phenylindole (DAPI), and allowed to cure overnight, at 4 °C in the dark, before being sealed and imaged.

Additional cryosections from young and old SDFTs and CDETs (n = 2) were immunostained simultaneously for both elastin (mouse monoclonal, ab9519, Abcam Cambridge, UK) and the endothelial cell marker CD31 (rabbit polyclonal, ab28364, Abcam Cambridge, UK;1:25 dilution). The same protocol described above was adopted. However, instead of methanol, 4% paraformaldehyde was used to fix the sections. Alexa Fluor 555 Goat anti-Mouse IgG and Alexa Fluor 488 Goat anti-Rabbit IgG were used as secondary antibodies for elastin and CD31, respectively.

Negative controls, in which the primary antibody was omitted from the blocking buffer, were included. A mouse IgG1 (M5284; 1:100 dilution) isotype control was also included. No staining was observed in any negative control samples, which confirms no nonspecific staining from the primary or secondary antibodies (Supplementary Figs 2 and 3, respectively). Transverse cryosections of the equine palmar common digital vein from young and old horses (n = 2) were also stained as positive controls. Sections were stained for elastin, CD31 and DAPI using the same protocol described above (Supplementary Fig. 4).

Imaging was performed on a confocal microscope (Leica TCS SP2) using × 40 (oil) objective. For each section, at least 2 regions were imaged in an area where both fascicular and interfascicular regions could be visualised. Brightfield images were used to identify the IFM and FM. Images were captured at a resolution of 1024 × 1024 pixels.

#### Image analysis

Semi-quantitative analysis of the confocal images was performed using ImageJ software. Fascicular matrix (FM) and interfascicular matrix (IFM) areas were identified based on their appearance under bright-field microscopy and UV fluorescence, where cell morphology and density, alongside collagen organisation were adopted as determining factors to distinguish the two regions (Supplementary Fig. 5). The amount of elastin in each region was determined in a semi-quantitative manner by calculating the percentage area stained positive for elastin. Additionally, the ratio of IFM:FM area was also determined for each image, enabling a comparison of the area occupied by IFM between tendon types and how it changes with ageing.

#### Fast Fourier Transform

**Figure 7:**
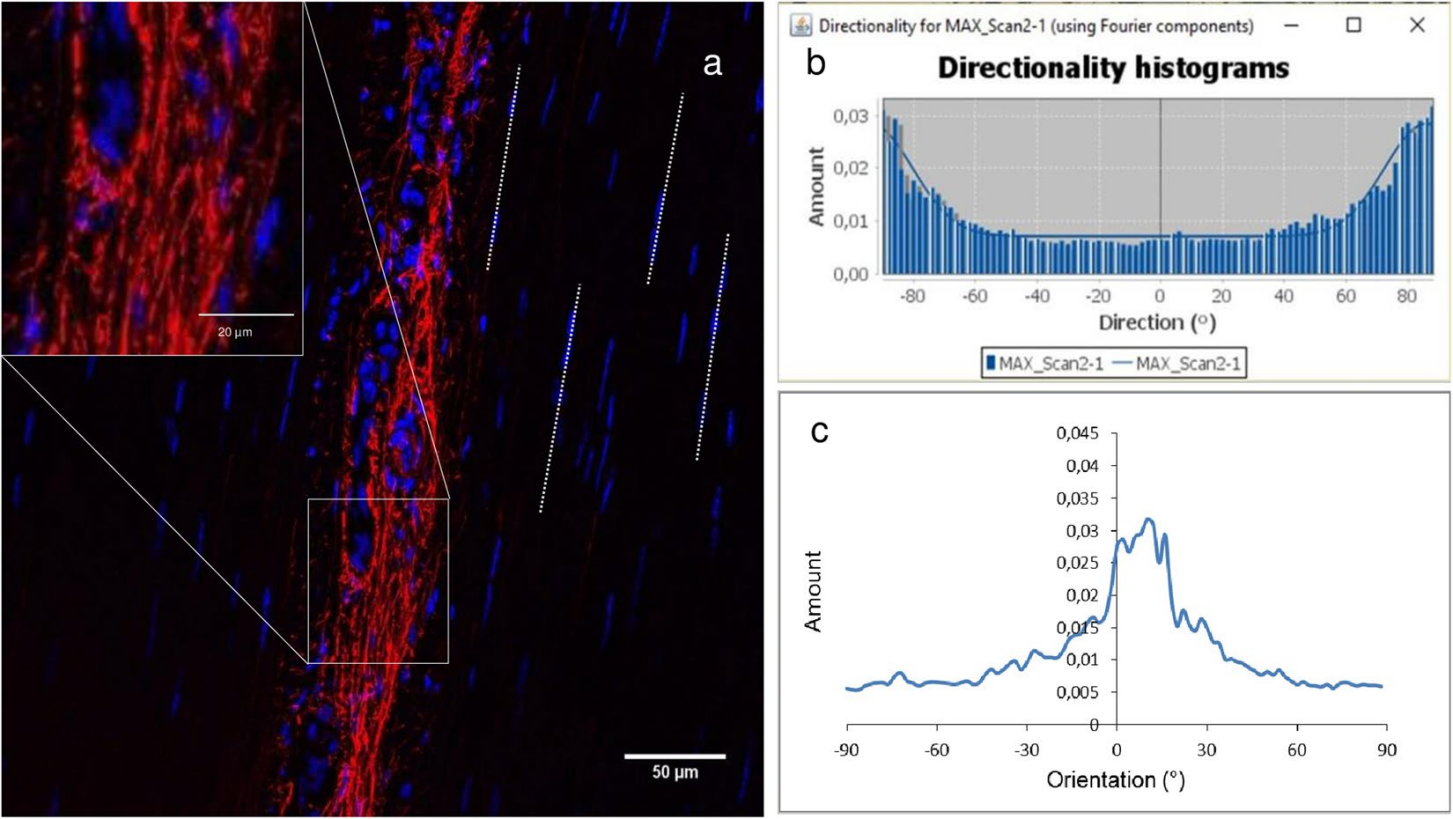
Pictorial representation of the method used to determine the distribution of elastin fibre alignment within histological sections. The orientation of the tendon long axis was first established. Four lines were drawn connecting rows of tenocytes within the tendon fascicle and, from these, an average orientation was calculated and adopted as the tendon long axis (**a**). The orientation of elastin fibres within the IFM (seen more clearly in the inset) was then determined relative to the tendon long axis. The image J Directionality plugin was then run, to give frequency distribution histograms, indicating the number of fibres in any given direction, initially relative to the image x axis, reporting the data in bins of 2 degrees over an angular range of 180 degrees (−90 to +90 degrees, where 0 is aligned with the x axis) (**b**). Taking the calculation of tendon long axis, described in part a, fibre angles were corrected, such that 0 degrees would correspond to the direction of the tendon long axis, and the frequency distribution redrawn (**c**).

Confocal images were also analysed using a Fast Fourier Transform (FFT), which allows the extraction of quantitative data regarding elastin fibre orientation and alignment^37^. The orientation of the tendon long axis was first defined from the ordered structure of fascicular matrix, as demonstrated in Fig. 7b. Four lines were drawn connecting the rows of tenocytes within the tendon fascicle, and from these, the average fascicle orientation established, and adopted as the tendon long axis. The directionality plugin was run in Fiji/ImageJ^53^ which, having performed a FFT on the original image, determines the preferred orientation of structures present in the input image relative to the long axis. This plugin computes a frequency distribution histogram, from the transformed image, indicating the number of structures in a given direction. Briefly, it divides the image into small squares and computes their Fourier power spectra. Later, these are analysed in polar coordinates and the power is measured for each angle^37^. Data were reported in bins of 2 degrees over an angular range of 180 degrees (−90 to +90 degrees), with zero degrees denoting alignment with the tendon long axis. The circular standard deviation was calculated for each image, using CircStat (MATLAB) which returns the circular standard deviation of a vector of circular data. The mean and standard deviation of the circular standard deviation were determined for each tendon type and age group. To facilitate data visualisation, values were grouped together to generate 9 bins (20 degrees each) (Fig. 5).

#### Image scoring

The same confocal images were scored semi-quantitatively for elastin organisation by four independent scorers, blinded to tendon type and age. The observers scored overall elastin organisation, and elastin fibre alignment parallel and perpendicular to the IFM. For each image, overall elastin organisation within the IFM was scored on a scale from 1 to 5, where 1 denoted highly disorganised elastin and 5 denoted highly organised elastin. Looking more specifically at the orientation of elastin in the IFM, elastin alignment parallel or perpendicular to the IFM was also scored on a scale from 1–5, where 1 was used to denote poor alignment in the direction of interest and 5 denoted high alignment in that direction. Inter observer agreement was assessed by using an online software tool http://dfreelon.org/utils/recalfront/recal3/ which determines the average pairwise Cohen’s Kappa statistic^33^.

### Statistical Analysis

All statistical analysis was carried out using the software *Minitab 17* for Windows.

All data are shown as mean ± standard deviation. Data were tested for normality using the Anderson – Darling test. Having established the normality of all data sets, two-way ANOVA (grouping information using the Tukey Method and 95% confidence) was used to evaluate differences in elastin content and desmosine crosslinking between tendon types and age groups. Two-way ANOVA were also used to determine differences in the variables reported from the image analysis and elastin semi-quantification. Two sample unpaired t-tests were adopted to determine differences in circular standard deviation. The results were considered statistically significant for p < 0.05.

### Data availability

The datasets generated and/or analysed during the current study are available from the corresponding author on reasonable request.

## Electronic supplementary material

Supplementary Information
