# Supplementary material for: Elastin is Localised to the Interfascicular Matrix of Energy Storing Tendons and Becomes Increasingly Disorganised With Ageing
Source: Sci Rep. 2017 Aug 30;7:9713. doi: 10.1038/s41598-017-09995-4 (PMC5577209; doi:10.1038/s41598-017-09995-4)
Supplement: Supplementary file 1 — Supplementary Information [file 41598_2017_9995_MOESM1_ESM.pdf]

Elastin is Localised to the Interfascicular Matrix of Energy Storing Tendons and Becomes Increasingly Disorganised With Ageing: Supplementary Information

Marta S. Godinho[1], Chavaunne T. Thorpe[2], Steve E. Greenwald[3], Hazel R. Screen[1*]

[1] Institute of Bioengineering, School of Engineering and Materials Science, Queen Mary University of London, London, E1 4NS, United Kingdom

[2] Comparative Biomedical Sciences, The Royal Veterinary College, Royal College Street, London, NW1 0TU, United Kingdom

[3] Blizard Institute, Barts and London School of Medicine and Dentistry, Turner Street, London E1 11BB, United Kingdom

*Corresponding Author:

Hazel R. C. Screen, PhD

School of Engineering and Materials Science,

Queen Mary University of London,

London, E1 4NS, United Kingdom

h.r.c.screen@qmul.ac.uk

Supplementary Figure Legends

Supplementary Figure 1. Representative images of a single longitudinal section of the SDFT showing elastin (red) (a) and CD31 (green) (b) staining as isolated channels. The combined elastin and CD31 staining can be seen in image c. Sections were also stained with DAPI (blue) and this channel of information is additionally shown in (d). Scale bar= 50μm.

Supplementary Figure 2. Representative image of the isotype control performed on a longitudinal section of the SDFT showing no non-specific staining of primary antibody used. Scale bar= 50μm.

Supplementary Figure 3. Representative image of the negative control, in which the primary antibody was omitted, performed on a longitudinal section of the SDFT showing no non-specific staining of secondary antibody used. Scale bar= 50μm.

Supplementary Figure 4. Representative image of the positive control performed on a transverse section of the equine palmar common digital vein showing the organisation of elastin (red) and CD31 (green). Scale bar= 200μm. Endothelial cells in small vessels embedded in the media and adventitia of the vein are clearly seen.

Supplementary Figure 5. Representative image of a longitudinal section of the SDFT showing tendon structure and cell morphology used to identify the IFM, which is enclosed by white dashed lines. Note the greater cell density, and more rounded morphology of cells, in the IFM compared to those in the FM. Scale bar= 50μm.

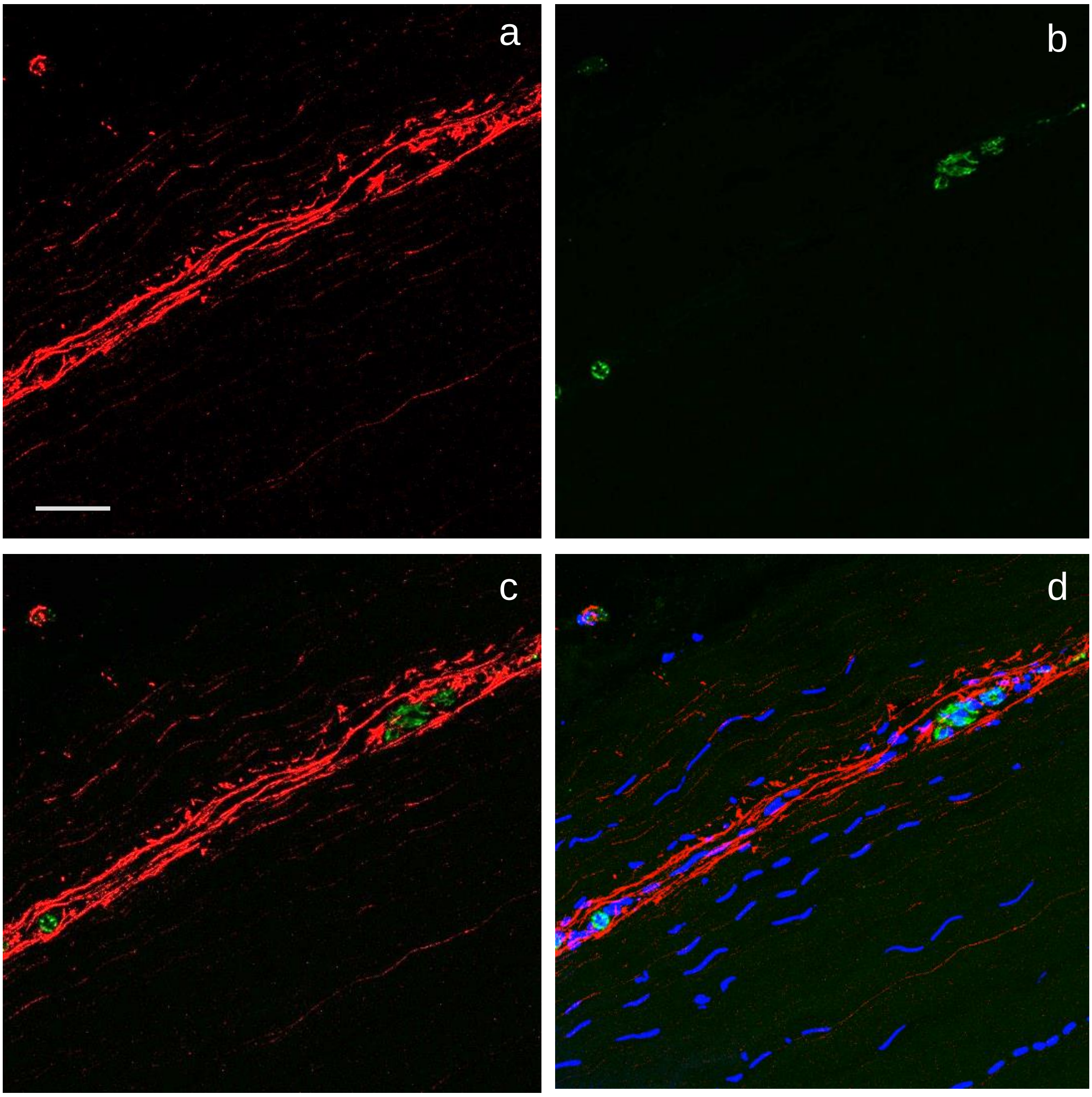

Supplementary Figure S1. Representative images of a single longitudinal section of the SDFT showing elastin (red) (a) and CD31 (green) (b) staining as isolated channels. The combined elastin and CD31 staining can be seen in image c. Sections were also stained with DAPI (blue) and this channel of information is additionally shown in (d). Scale bar= 50μm.

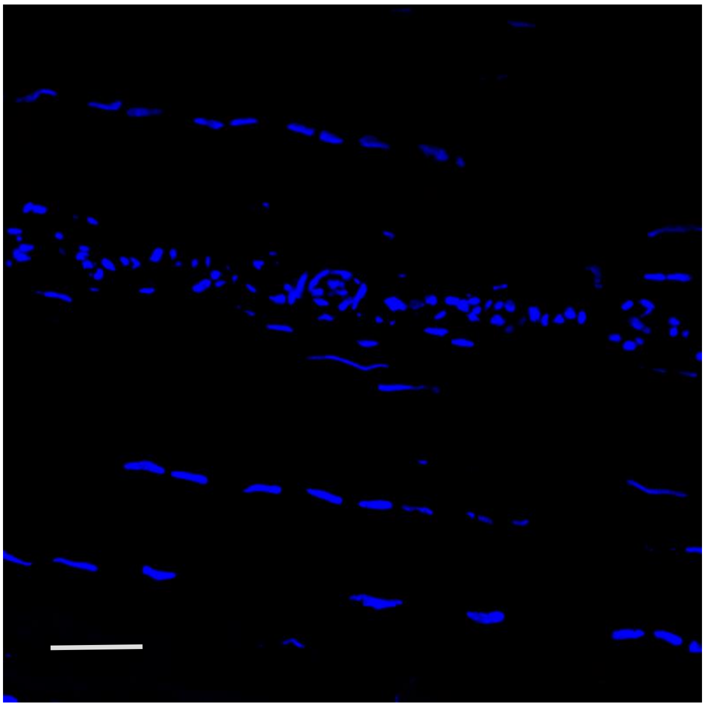

Supplementary Figure S2. Representative image of the isotype control performed on a longitudinal section of the SDFT showing no non-specific staining of primary antibody used. Scale bar= 50μm.

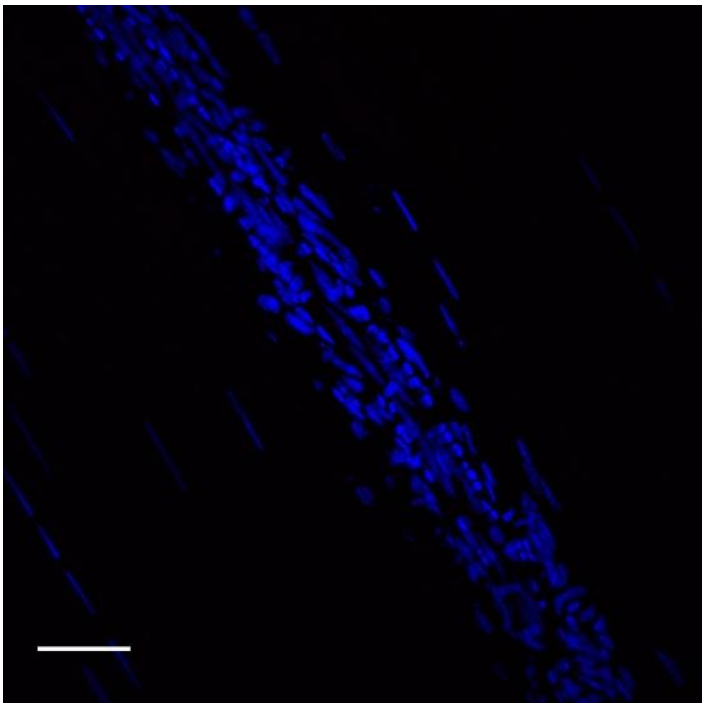

Supplementary Figure S3. Representative image of the negative control, in which the primary antibody was omitted, performed on a longitudinal section of the SDFT showing no non-specific staining of secondary antibody used. Scale bar= 50µm.

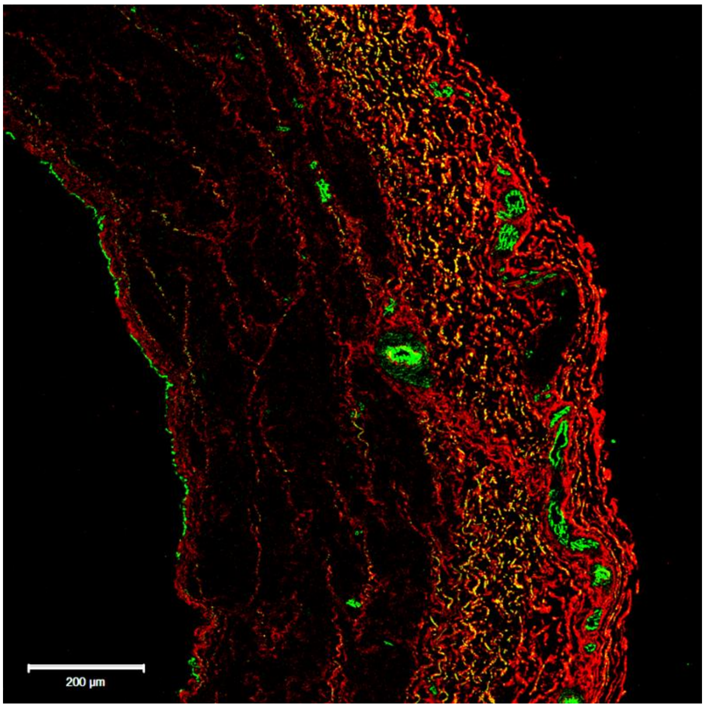

Supplementary Figure S4. Representative image of the positive control performed on a transverse section of the equine palmar common digital vein showing the organisation of elastin (red) and CD31 (green). Scale bar= 200µm. Endothelial cells in small vessels embedded in the media and adventitia of the vein are clearly seen.

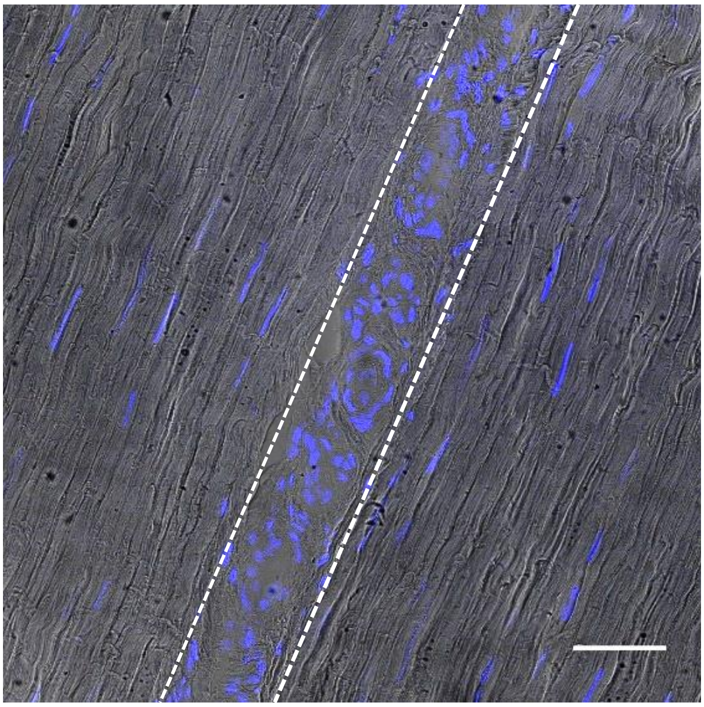

Supplementary Figure S5. Representative image of a longitudinal section of the SDFT showing tendon structure and cell morphology used to identify the IFM, which is enclosed by white dashed lines. Note the greater cell density, and more rounded morphology of cells, in the IFM compared to those in the FM. Scale bar= 50µm.
